# Correcting mistakes in predicting distributions

**DOI:** 10.1093/bioinformatics/bty346

**Published:** 2018-05-14

**Authors:** Valérie Marot-Lassauzaie, Michael Bernhofer, Burkhard Rost

**Affiliations:** Department of Informatics, l12–Chair of Bioinformatics and Computational Biology, Technical University of Munich (TUM), Garching/Munich, Germany

## Abstract

**Motivation:**

Many applications monitor predictions of a whole range of features for biological datasets, e.g. the fraction of secreted human proteins in the human proteome. Results and error estimates are typically derived from publications.

**Results:**

Here, we present a simple, alternative approximation that uses performance estimates of methods to error-correct the predicted distributions. This approximation uses the confusion matrix (TP true positives, TN true negatives, FP false positives and FN false negatives) describing the performance of the prediction tool for correction. As proof-of-principle, the correction was applied to a two-class (membrane/not) and to a seven-class (localization) prediction.

**Availability and implementation:**

Datasets and a simple JavaScript tool available freely for all users at http://www.rostlab.org/services/distributions.

**Supplementary information:**

Supplementary data are available at *Bioinformatics* online.

## 1 Introduction

Proteome-wide distributions of biological characteristics are relevant for many experimental and computational tools. Two examples pertinent to asses experiments and resources are: what fraction of the proteins are enzymes? What fraction of sequence variants strongly affects function? Often neither experimental annotations nor computational predictions infer the true distribution.

Here, we introduced a simplified approximation to compensate bias. To implement this approximation, users only need the confusion matrix describing the performance of a method along with predictions for entire proteomes or more generally datasets.

## 2 Materials and methods

### 2.1 Error correction approximation

First, look up the confusion matrix, i.e. the matrix (or table) describing the evaluation of a method. For two class-predictions these are typically the true positives (TP), true negatives (TN), false positives (FP) and false negatives (FN). The matrix *M* with elements *M_p, o_* gives the number of proteins predicted in class *p* and observed in class *o*. Correct predictions are diagonal *o *=* p*. M has the dimension *n* (number of classes), e.g. *n *=* *2 for distinguishing secreted from non-secreted proteins. From this matrix follows a new n*n matrix *M′* representing the confusion ratio for each class with:

(1) $$M\prime_{p,\;o}=\frac{M_{p,o}}{\sum_{i=1}^{n}M_{p,i}}$$

Each value *M′*_*p, o*_ in this new matrix represents the ratio of events predicted in class *p* and observed as *o* over all events predicted in class *p*. From this matrix predictions for an entire proteome/dataset *P* = (*p_1_*, *p_2_*,…, *p_n_*) are corrected to *P_c_* = (*c_1_*, *c_2_*,…, *c_n_*) by:

(2) $$c_{x}=\sum_{i=1}^{n}M{'}_{i,x}*p_{i}$$

Each value *c_x_* represents the number of events predicted in class *x* multiplied by the ratio of events correctly predicted as *x* (real *x*) added to the number of events predicted in every other class multiplied by the confusion rate of this other class to *x*.

### 2.2 Datasets and prediction methods

Two datasets with experimentally determined localization were used. (i) The set with all 5563 human protein annotations from Swiss-Prot (Pundir *et al.*, 2017) was used to build the confusion matrices for the methods. (ii) All high-confidence annotations were extracted from The Human Protein Atlas (Thul *et al.*, 2017) (HPA ‘Validated’ and ‘Supportive’) to evaluate the correction proposed [Equation (2)]. To avoid overlap, only proteins without Swiss-Prot annotations were added. This gave 2000 proteins. Three tools: Hum-mPloc3.0 (Zhou *et al.*, 2017), LocTree2 (Goldberg *et al.*, 2012) and MultiLoc2 (Blum *et al.*, 2009) were run to evaluate the error correction (Supplementary Table S1 for class conversion).

### 2.3 Distance between distributions

The error correction [Equation (2)] was used to compare 7-class distributions with and without error correction. The Euclidean distance (square root mean distance for all classes) served as proxy for the difference between the two.

## 3 Results and discussion

The approximation for the bias correction in experimental and computational data was applied to two problems: one was a hypothetical two-class prediction of the fraction of transmembrane proteins; the other illustrated a 7-class classification of protein localization. The correction was based on performance estimated using all experimental annotations for human in Swiss-Prot (Pundir *et al.*, 2017).

### 3.1 Mistakes may dominate for the non-optimized class

Many methods optimize the prediction of membrane helices. Those typically focus on membrane proteins. Assume such a method misses less than 1% of all proteins with membrane helices. Assume the same method incorrectly finds membrane regions in 10% of the non-membrane proteins. Organism *H* might have 20 000 (20k) proteins, with 5k transmembrane. The prediction method would make 1.5k mistakes in the 15k non-membrane (10% error) and 50 in the membrane proteins. Thus, instead of finding 25% as membrane, methods would suggest ∼32% (5000–50 + 1500∼6450/20000). The error corrected version would find the correct number 25%. The extreme imbalance in performance between the class optimized (membrane) and the other (non-membrane) is not unusual for prediction methods (Chen *et al.*, 2002; Reeb *et al.*, 2015).

### 3.2 Distributions approximated better after correction

The error correction was benchmarked on a multi-class problem using 2000 new proteins with experimental 7-class annotations from HPA (Thul *et al.*, 2017). The problem (predict 7-class distribution) was particularly interesting because the correct distribution (HPA) differed substantially from data used for method development, i.e. all methods optimized a different distribution. On top, none of today’s experimental distributions might capture the entire proteome.

**Fig. 1.**
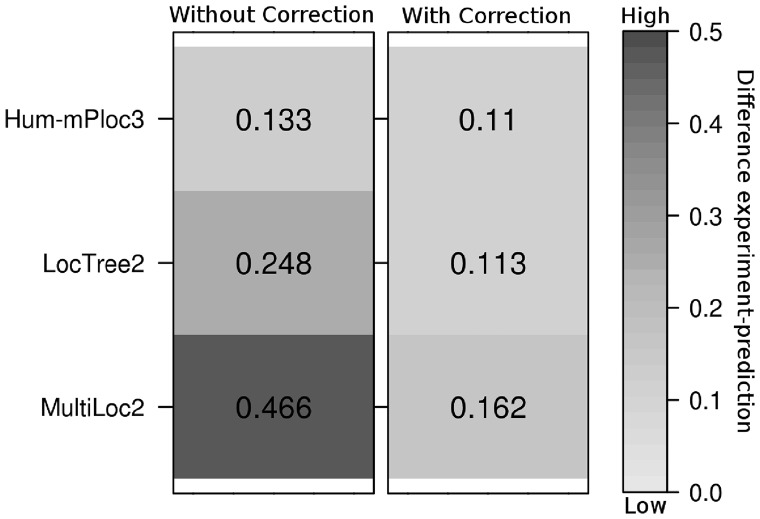
Better estimates of location distribution through error correction. Values give the Euclidean distance between 7-class distributions from experiment (The HPA) and those predicted directly (left) and predicted with error correction approximation (right)

The comparison between the distributions generated from the raw method output (Fig. 1, left panel) and through the error correction [Equation (2); Fig. 1, right panel] clearly showed a substantial improvement (lighter means more correct). Our approximation optimized no parameter to succeed. It also requires only information made available by the developers. An interesting effect of the error-correction is that the best methods might not always give the best error-corrected distribution (Supplementary Fig. S2).

## 4 Conclusion

The simplified approximation [Equation (2)] for correcting the bias in experimental and computational views on complete datasets (e.g. entire proteome or genome, all patients in a region) is neither limited to two classes (Fig. 1), nor to proteins, genes, or any other omics. Instead, it is applicable to all datasets that estimate distributions for any aspect. The correction only requires that the confusion matrix reflecting the performance of a method (computational or experimental) correctly reflects unseen data. Nevertheless, our example also showed that methods might use different distributions and still improve substantially through applying the approximation.

## Supplementary Material

Supplementary Data
