# Supplementary material for: Correcting mistakes in predicting distributions
Source: Bioinformatics. 2018 May 14;34(19):3385–6. doi: 10.1093/bioinformatics/bty346 (PMC6157078; doi:10.1093/bioinformatics/bty346)
Supplement: Supplementary Data [file bty346_suppl_data.doc]

Supporting Online Material (SOM) for:
*Correcting mistakes in predicted distributions*

Valérie Marot-Lassauzaie, Michael Bernhofer & Burkhard Rost

# Short description of SOM

This file contains the Supporting Online Material for the publication “Correcting mistakes in predicted distributions”.

Table S1 provides the details how the output from the three prediction tools and from The Human Protein Atlas [1] was converted to a set of seven “main” localization classes for the comparison of the output.

Figure S2 adds another test to illustrate that the proposed correction method can improve predicted distributions. The predictions are compared to the experimentally established standard-of-truth for a subset of 1315 human proteins for which annotations were extracted from the scientific literature using the text mining tool LocText[2].

# SOM

**Table S1: Conversion of annotations to seven localization classes*.**

| *Localization class \ Tool* | *The Human Protein Atlas [1]* | *MultiLoc2 [3]* | *Hum-mPloc3.0 [4]* | *LocTree2 [5]* |
| --- | --- | --- | --- | --- |
| Secreted | N/A | Extracellular | Extracellular | Extracellular |
| Nucleus | nucleoplasm, nuclear bodies, nuclear speckles, nucleus, nucleoli, nucleoli fibrillar centre, nuclear membrane | Nucleus | Nucleus | Nucleus, nucleus membrane |
| Cytoplasm | Cytosol, microtubule, microtubule ends, microtubule organising centre, intermediate filaments, cytoplasmic bodies, actin filaments, centrosome, midbody, cytokinetic bridge, mitotic spindle, rods & rinds, midbody ring | Cytoskeleton, Cytoplasmic | Centriole, Cytoplasm, Cytoskeleton | Cytosol |
| Plasma membrane | Plasma membrane, Focal adhesion sites, cell junction | Plasma membrane | Plasma membrane | Plasma membrane |
| Mitochondrion | Mitochondrion | Mitochondrion | Mitochondrion | Mitochondria, mitochondria membrane, chloroplast^**^, chloroplast membrane^**^, plastid^**^ |
| Endoplasmic reticulum | Endoplasmic reticulum | Endoplasmic reticulum | Endoplasmic reticulum | Endoplasmic reticulum, endoplasmic reticulum membrane |
| Golgi apparatus | Golgi apparatus | Golgi apparatus | Golgi apparatus | Golgi apparatus,  Golgi apparatus membrane |

* To compare *in silico* predictions and experimental annotations, we needed to compare all methods based on the same number of localization classes. Toward this end, we chose 7 classes and converted the output of the prediction tools (MultiLoc2 [3], LocTree2 [5], Hum-mPloc3.0 [4]) and from The Human Protein Atlas [1] as follows. Any entry not fitting into any of the seven classes was excluded from comparison.

** LocTree2 [5] published: “chloroplast and plastid classes are valid for plant proteins only; if the origin of a non-plant protein is known, please consider prediction of these classes as mitochondrial”

**Fig. S2: Evaluation of the error-correction method on another dataset.** The method for the correction of mistakes in predicted distributions has been evaluated against a second data set of experimental annotations for 1315 human proteins in Swiss-Prot extracted from scientific literature through the text mining tool LocText [2]. As for Fig. 1 in the main manuscript, the values shown reflect the Euclidean distance between 7-state distributions from experiment (LocText) and those predicted directly (left) and predicted with the error correction approximation (right). For this particular subset of proteins, the method performing best before error correction (Hum-mPloc3) no longer performs best after error correction, although even for this method the error correction clearly improved the estimate.


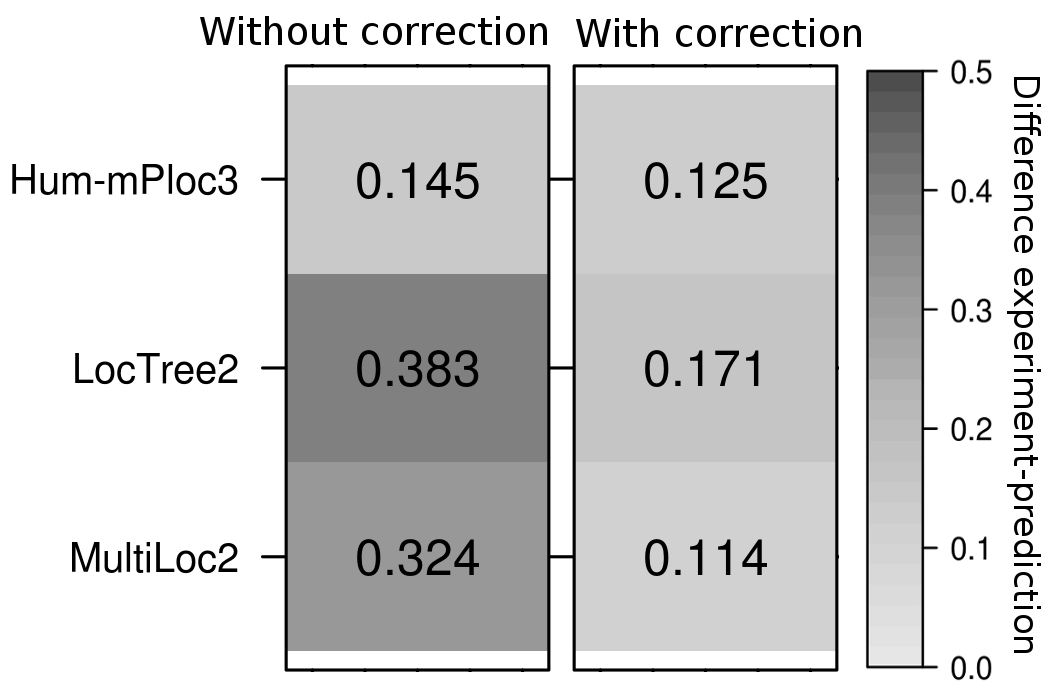


# References for Supporting Online Material

1. Thul, P.J., et al., *A subcellular map of the human proteome.* Science, 2017. **356**(6340).

2. Cejuela, J.M., et al., *LocText: relation extraction of protein localizations to assist database curation.* BMC Bioinformatics, 2018. **19**(1): p. 15.

3. Blum, T., S. Briesemeister, and O. Kohlbacher, *MultiLoc2: integrating phylogeny and Gene Ontology terms improves subcellular protein localization prediction.* BMC Bioinformatics, 2009. **10**: p. 274.

4. Zhou, H., Y. Yang, and H.B. Shen, *Hum-mPLoc 3.0: prediction enhancement of human protein subcellular localization through modeling the hidden correlations of gene ontology and functional domain features.* Bioinformatics, 2017. **33**(6): p. 843-853.

5. Goldberg, T., T. Hamp, and B. Rost, *LocTree2 predicts localization for all domains of life.* Bioinformatics, 2012. **28**(18): p. i458-i465.
